# Prediction and Severity Ratings of COVID-19 in the United States

**DOI:** 10.1017/dmp.2020.343

**Published:** 2020-09-10

**Authors:** Liping Yue, Taotao Tu, Xiuyuan Geng

**Affiliations:** College of Economics and Management, Huazhong Agricultural University, Wuhan, Hubei Province

**Keywords:** COVID-19, prediction, severity rating

## Abstract

**Objectives:**

The objectives of this study is to predict the possible trajectory of coronavirus disease 2019 (COVID-19) spread in the United States. Prediction and severity ratings of COVID-19 are essential for pandemic control and economic reopening in the United States.

**Method:**

In this study, we apply the logistic and Gompertz model to evaluate possible turning points of the COVID-19 pandemic in different regions. By combining uncertainty and severity factors, this study constructed an indicator to assess the severity of the coronavirus outbreak in various states.

**Results:**

Based on the index of severity ratings, different regions of the United States are classified into 4 categories. The result shows that it is possible to identify the first turning point in Montana and Hawaii. It is unclear when the rest of the states will reach the first peak. However, it can be inferred that 75% of regions will not reach the first peak of coronavirus before August 2, 2020.

**Conclusion:**

It is still essential for the majority of states to take proactive steps to fight against COVID-19 before August 2, 2020.

The coronavirus disease 2019 (COVID-19) pandemic has caused major disruption to the global economy. According to Centers for Disease Control and Prevention (CDC), the total number of confirmed COVID-19 cases reached 3,296,599 on July 13, 2020, in the United States, which ranks first in the world.^1^ On March 16, 2020, San Francisco began to adopt a lockdown measure, which aims to contain the spread of coronavirus. After that, most states have imposed similar measures restricting gathering and social contact. The lockdown measures are essential for virus containment. However, they hurt people’s livelihoods and brought chaos to the labor market and economic activity in the United States. According to the US Bureau of Labor Statistics, total nonfarm payroll employment fell by 2,050,000 in April 2020, and the unemployment rate rose to 14.7%.^2^ The coronavirus epidemic has devastated the national economy, with some 30 million Americans seeking unemployment benefits between March 21 and April 30. The economic forecasting company Goldman Sachs predicted that the US gross domestic product (GDP) will fall by 3.8% in 2020, with growth falling 24% in the second-quarter GDP, and the unemployment rate expected to rise to 3.5-9% in the coming quarters.^3^ According to a report released by Congressional Budget Office (CBO), real (inflation-adjusted) consumer spending fell by 17% from February to April, as social distancing reached its peak. In April, car and light truck sales were 49% below the 2019 monthly average. Mortgage applications fell by 30% in April 2020 compared with the same period in 2019.^4^

Loosening coronavirus measures and reopening the economy have become the top priority for many states. However, to ease restrictions and resume business, it is crucial to know when COVID-19 cases in the United States will peak. Therefore, it makes sense to predict coronavirus trajectories in different states.

Review of the literature shows that the compartment model is the most widely used model, which can predict the characteristics of disease spread, such as the degree of the epidemic, and the duration of the outbreak. The susceptible-infected-recovered (SIR) model is one of the simplest compartment models, which has 4 underlying assumptions.^5^ First, the total population of a city is constant. Second, the health status of the community can be divided into 3 categories, namely susceptible, infected, and recovered. Third, the population is immobile, ie, no patients flow into or out of the city. Fourth, infected patients under strict isolation will no longer infect others. The SIR model can compute the theoretical number of people infected with coronavirus in a closed population over time.^6^ However, SIR is only applicable to some acute infectious diseases that do not have an incubation period. To overcome this limitation, the susceptible-exposed-infectious-recovered (SEIR) model further takes into account a group of people who have the disease but are not yet infectious. The SEIR model enables analysis of disease spread, taking into account incubation periods.^7^ By adding a group of people with maternally derived passive immunity and another group having the disease but not yet infectious, SEIR model can be extended into an immunity-susceptible-exposed-infectious-recovered (MSEIR) model.^8^

It should be pointed out that the accuracy of the prediction relies on the basic reproduction number R0 for the above models.^9^ The basic reproduction number, R0, is the most critical parameter to determine the intrinsic transmissibility, which is the average number of secondary infectious cases generated by an index case in a completely susceptible population without any interventions.^10^ During the outbreak of an epidemic, the actual R0 is dynamic, which depends on the containment measures and governance patterns. For example, studies have found that R0 is different at various stages of the coronavirus epidemic (Table 1).^11-15^ Therefore, it is entirely reasonable to obtain different predictions of R0 for various research (Table 1).

**TABLE 1:** Prediction of the Basic Reproduction Number of COVID-19

Author	Country	Sample Period	Data sources	R0
Pan et al.^11^	China	December 2019 to March 8,2020	Municipal Notifiable Disease Report System	0.3-3
Zhao et al.^12^	China	January 10 to January 24,2020	Wuhan Municipal Health Commission and National Health Commission of China	2.24-3.58
Torres-Roman et al.^13^	Peru	March 6 to March 15,2020	Peruvian Ministry of Health	2.97
Zhuang et al^14^	Korea	January31 to March 5,2020	Public Information Calculation	2.5 and 3.2
	Italy	February 5 to March 10,2020	National Statistics porta	2.6 and 3.3
Choi and Ki^15^	Korea	January 20 to March 4,2020	Korea Centers for Disease Control and Prevention	0.55

Because R0 is changing in different stages of the outbreak, it may be difficult to make a precise prediction on the spread of infectious disease using compartment models. To avoid the difficulties of R0 estimation, we intend to use the logistic and Gompertz models to predict coronavirus trajectories in different states. One advantage of the 2 types of models is that they can make full use of the historical data of coronavirus cases, thus taking into account the real-time information of the infection number.^16^ Based on the prediction result of the 2 models, we aim to put forward a methodology to identify the turning point in the spread of coronavirus in the United States. We will also introduce a grading index to measure the severity ratings of COVID-19 in different regions of the United States.

## METHODS

### Identification of the Turning Point

**FIGURE 1:**
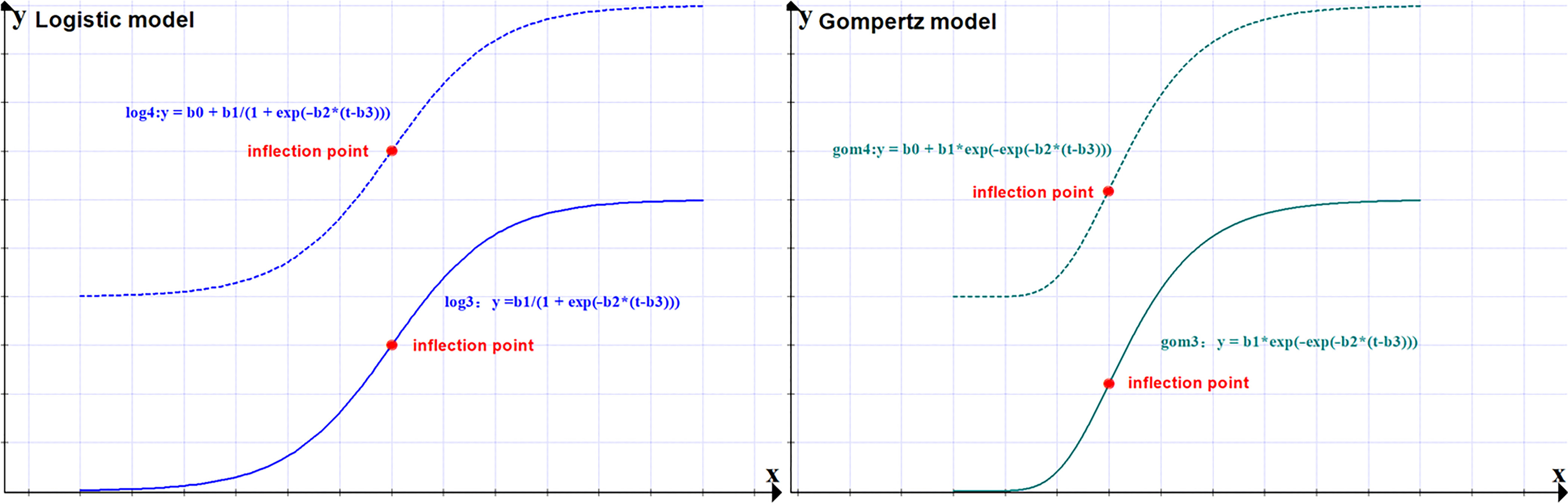
Logistic and Gompertz curve.

To avoid the prediction difficulties caused by fluctuation of the basic reproduction number R0, we use the logistic and Gompertz function to predict confirmed COVID-19 cases in the United States. To increase the robustness of prediction, we will apply 4 models based on logistic and Gompertz functions (see Table 2). A logistic function is a typical “S” shape (sigmoid curve), while the Gompertz function is an asymmetric sigmoid shape (see Figure 1). In other words, the logistic model has the property of being symmetrical about the inflection point, while Gompertz curve is asymmetrical about the inflection point.^17^

**TABLE 2:** Logistic and Gompertz Functions

Name of the Model	Function Mefinition
log4	y = b0 + b1/(1 + exp(-b2*(t-b3)))
log3	y = b1/(1 + exp(-b2*(t-b3)))
gom4	y = b0 + b1*exp(-exp(-b2*(t-b3)))
gom3	y = b1*exp(-exp(-b2*(t-b3)))

In logistic and Gompertz functions, y represents the evolution of the epidemic in the United States, which is the cumulative confirmed cases of COVID-19. t stands for time. Both log4 and log3 are logistic functions: log4 is a logistic function with intercept term b0, and log3 is the logistic function without an intercept term. Both gom4 and gom3 are Gompertz functions: gom4 is the Gompertz function with intercept term b0, and gom3 is the Gompertz function without an intercept term. b1 is the curve’s maximum value. b2 is the steepness of the curve. b3 is the t-value of the sigmoid’s midpoint.

The trajectories predicted by 4 models represent 4 paths that may appear in COVID-19’s transmission. As external conditions change, the cumulative number of confirmed cases may follow either trajectory. Examining the peaks of the 4 paths will help identify the turning point of COVID-19 in the United States.

Based on the 4 models in Table 1, *path*_*log4*_, *path*_*log3*_, *path*_*gom4*_, and *path*_*gom3*_ are the 4 predicted trajectories. Suppose 4 paths reached their peak at time T1, T2, T3, and T4, respectively, which needs to meet the following conditions.(1)
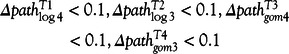


The meaning of constraint Equation (1) is that the daily increase of confirmed cases at the peak must be less than 0.1. In other words, the cumulative confirmed cases of COVID-19 at the peak of 4 predicted trajectories would remain stable. However, it is difficult to claim that the pandemic will reach a maximum if there is significant divergence among T1, T2, T3, and T4. To confirm the turning point for a specific state in the United States, the 4 predicted trajectories need to converge. After identifying T1, T2, T3, and T4 based on Equation (1), the time interval between the turning point of the highest path and the lowest path is defined as follows.(2)
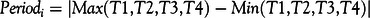


Therefore, the following constraint must hold for an identified turning point.(3)
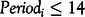


When the constraint Equation (3) holds, the time interval between the most pessimistic and optimistic turning points is less than or equal to 2 wk. Then it can be inferred that the 4 predicted trajectories would converge under constraint Equation (3). As a result, we can claim with confidence that the identified turning point lies between *Min*(*T*1, *T*2, *T*3, *T*4) and *Max*(*T*1, *T*2, *T*3, *T*4). If the constraint Equation (3) does not hold, we can conclude that it is still too early to determine the turning point based on the historical data of cumulative confirmed cases of COVID-19. However, it should be mentioned that the models used here rely on a closed nonmobile population. Since the death of George Floyd, the Black Lives Matter movement, which began on May 26, has led to large scale protests in the United States. Therefore, the model might apply to the period before May 26.

### Severity Ratings of COVID-19

To rate the severity of COVID-19 spreading in the states, we take into account 2 crucial factors, namely the uncertainty of the outbreak and the gravity of the current circumstances. We collected data of COVID-19 outbreak in the United States from Wind Data Service, which is a market leader in China’s financial information services industry. We selected the sample period from January 21 to May 25, 2020, to make prediction, since Black Lives Matter protests across the United States happened after May 25. As noted before, the model used in this study depends on a closed nonmobile population. Thus, we apply the model to make estimation using the sample before May 26. To have a better view of the severity of COVID-19, turning points for 50 states and Washington D.C. are predicted.

### (1) Construction of Uncertainty Index

As is shown in Equation (2), *Period_i_* is the time interval between the turning point of the highest path and the lowest path, which represents the uncertainty of the epidemic trend. There will be less uncertainty about the epidemic trend if the value *Period_i_* is smaller. We further standardized them to eliminate the influence of the dimension as below.(4)
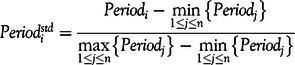


### (2) The Severity of the Epidemic

We use the number of existing confirmed cases to measure the severity of the current situation, which is defined as follows.(5)
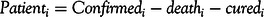


As is shown in Equation (5), the total number of existing confirmed cases in state i (*Patient_i_*) equals the cumulative number of confirmed cases (*Confirmed_i_*) minus the cumulative number of deaths (*death_i_*) and the cumulative number of recovered cases (*cured_i_*).The data provider of the 3 indicators is Wind Data Service. To minimize the effect of dimension, we calculated the proportion of existing confirmed cases as below.(6)
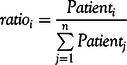


As is shown in Equation (6), *ratio_i_* can be used to measure the severity of the epidemic in state i. As *ratio_i_* becomes larger, coronavirus spread in state i is more severe.

### (3) Index of Severity Ratings for COVID-19

Combining information on uncertainty and severity, we construct the index of severity ratings as below.(7)
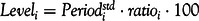


As mentioned above, a larger value of 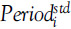 means greater uncertainty, while a larger *ratio_i_* means more severe of the epidemic in terms of existing confirmed cases. Therefore, as the index of severity rating *Level_i_* increases, the severity ratings for state i will rise accordingly. The severity index in Equation (7) takes into account both uncertainty and severity information, which can provide a reasonable evaluation of severity ratings for COVID-19 in the United States.

According to the estimation value of *Level_i_*, it is possible to divide different regions of the United States into 4 categories based on severity degree (Table 3). Using the information on first, second, and third quartile, we can identify regions with high risk, medium-high risk, medium-low risk, and low risk. To be more specific, if *Level_i_* lies between first and third quartile, then region i can be identified as medium risk areas. The second quartile is used to separate medium-high risk from medium-low risk areas. If *Level_i_* lies below first quartile or above third quartile, then region i can be identified as low risk area or high risk area.

**TABLE 3:** Severity Rating of COVID-19

Value of *Level_i_*	Severity Ratings
*Q*3 < *Level_i_*	High risk
*Q*2 < *Level_i_* < *Q*3	Medium-high risk
*Q*1 < *Level_i_* < *Q*2	Medium-low risk
*Level_i_* < *Q*1	Low risk

Note: Q1, Q2, Q3 are the first, second, and third quartile respectively. Inter-quartile range is the distance between the first and third quartiles.

Furthermore, by identifying outliers with the 1.5 × *IQR* rule, it is possible to distinguish extremely high-risk and extremely low-risk areas within high risk and low risk areas, respectively. To be more specific, if *Level_i_* is greater than *Q*3 + 1\hbox.5*IQR, then region i can be classified as extremely high-risk region within high risk group. If *Level_i_* is less than *Q*1 – 1.5*IQR, then region i can be classified as extremely low-risk areas within low risk group. There are 4 possibilities. First, there are no outliers for the index of severity rating. In this case, there is no extremely high-risk or extremely low-risk areas. Second, there might be only high outliers. In this case, we can distinguish extremely high-risk areas within high risk group. Third, there might be only low outliers. In this case, we can distinguish extremely low-risk areas within low risk group. Fourth, there might be both high and low outliers. In this case, we can distinguish both extremely high-risk and extremely low-risk areas within high risk group and low risk group, respectively.

## RESULTS

### Result Analysis

**FIGURE 2:**
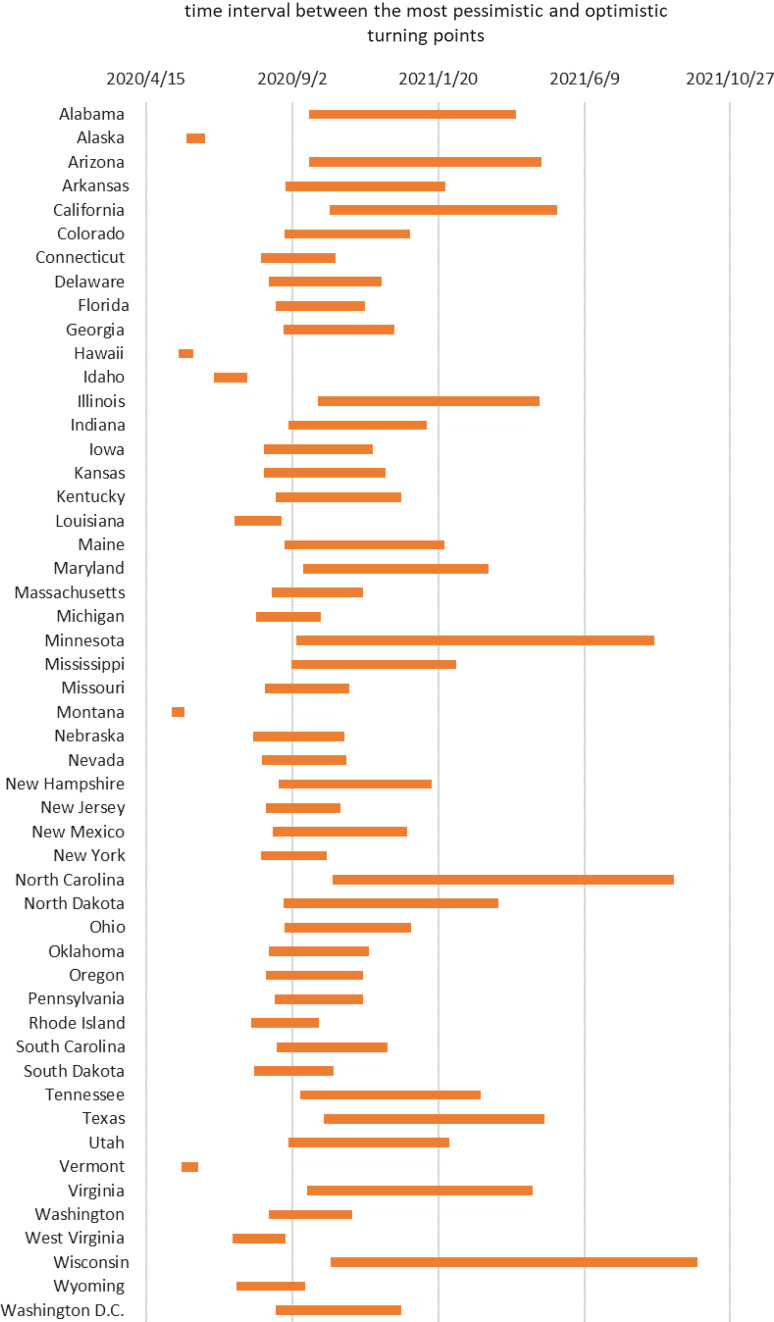
Time interval between the most pessimistic and optimistic turning points.

Using data of the number of confirmed cases from January 21, 2020, to May 25, 2020, in the United States, we derive the peak of 4 trajectories. As is shown in Equation (3), to identify a turning point, the time interval between the most pessimistic and optimistic turning points should be less than or equal to 14. Further analysis shows that only Montana and Hawaii satisfy this condition (see Figure 2). Therefore, it is only possible to identify the first turning point for the 2 states using the sample data. The first turning point of Montana lies between May 10, 2020, and May 22, 2020. And the first turning point of Hawaii lies between May 16, 2020, and May 30, 2020. For the rest of the regions of the United States, the time interval between the most pessimistic and optimistic turning points is larger than 2 wk. Thus, there is still great uncertainty of predicting the first turning point for other states. For instance, the estimated intervals for Arizona, California, Illinois, Minnesota, North Carolina, North Dakota, Texas, Virginia, and Wisconsin are larger than 200, as is shown in Figure 2. In other words, there is significant uncertainty of the COVID-19 spreading in the above 9 states. Although we cannot derive the exact first turning point for the majority of states, it can still be inferred from Figure 1 that 75% of regions will not reach the peak of coronavirus before August 2, 2020.

**FIGURE 3:**
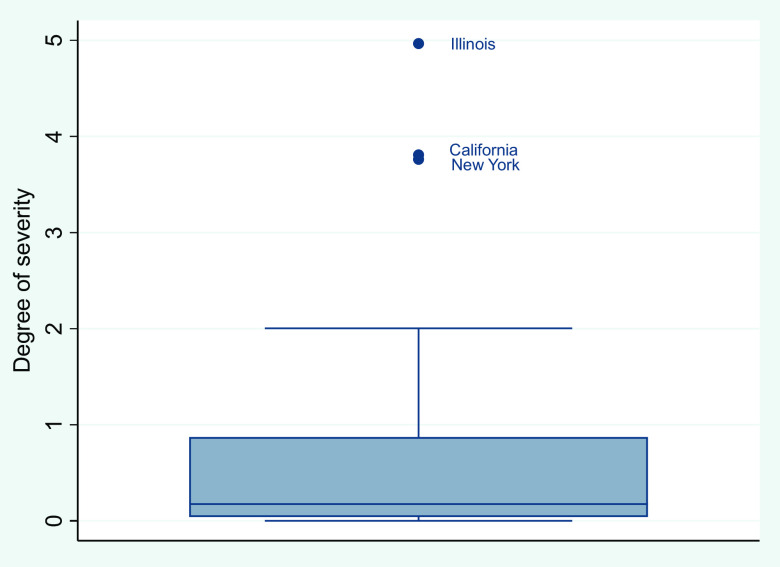
Box plot diagram to identify outliers.

**FIGURE 4:**
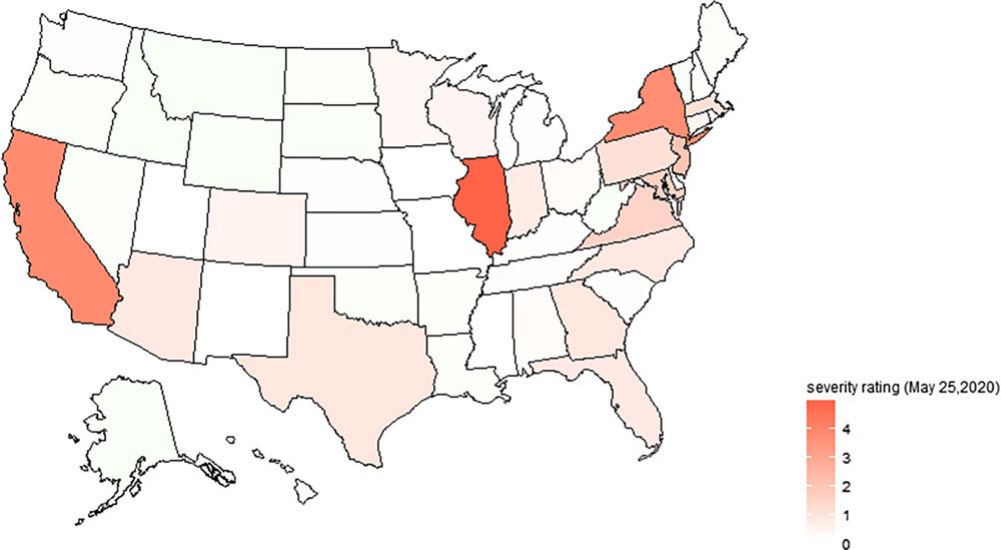
Severity rating of different regions in the United States.

Based on Equation (7), we derive the index of severity ratings for COVID-19. It is found that there are only high outliers, which include Illinois, California, and New York (see Figure 3). Therefore, among high-risk group, Illinois, California, and New York are extremely high-risk areas. As shown in Figure 4, the colors of different states are categorized according to the severity of the outbreak. As the color switches from green to red, severity ratings for COVID-19 will become more and more severe. We can see that Illinois, California, and New York have the deepest red colors; that is, the outbreak in these states are the most severe.

According to severity rating (see Table 4), there are 13 high-risk regions: Illinois, California, New York, New Jersey, Maryland, Virginia, Pennsylvania, Massachusetts, Texas, Georgia, Arizona, North Carolina, and Florida. Twelve states are medium-high risk areas: Indiana, Colorado, Connecticut, Wisconsin, Minnesota, Ohio, Alabama, Tennessee, Washington, Nebraska, Michigan, and Mississippi. Washington DC and 11 states are medium-low risk areas. Fourteen states are low-risk areas.

**TABLE 4:** Classification of Regions Based on the Severity Rating of COVID-19

Severity Ratings	Regions
High risk	Illinois, California, New York, New Jersey, Maryland, Virginia, Pennsylvania, Massachusetts, Texas, Georgia, Arizona, North Carolina, and Florida
Medium-high risk	Indiana, Colorado, Connecticut, Wisconsin, Minnesota, Ohio, Alabama, Tennessee, Washington, Nebraska, Michigan, and Mississippi
Medium-low risk	Washington D.C., Iowa, Rhode Island, New Mexico, Utah, Missouri, Kentucky, Kansas, South Carolina, Delaware, Arkansas, Louisiana
Low risk	New Hampshire, North Dakota, Oregon, Nevada, Maine, Oklahoma, South Dakota, West Virginia, Idaho, Wyoming, Vermont, Alaska, Hawaii, Montana

### Robustness Test

**FIGURE 5:**
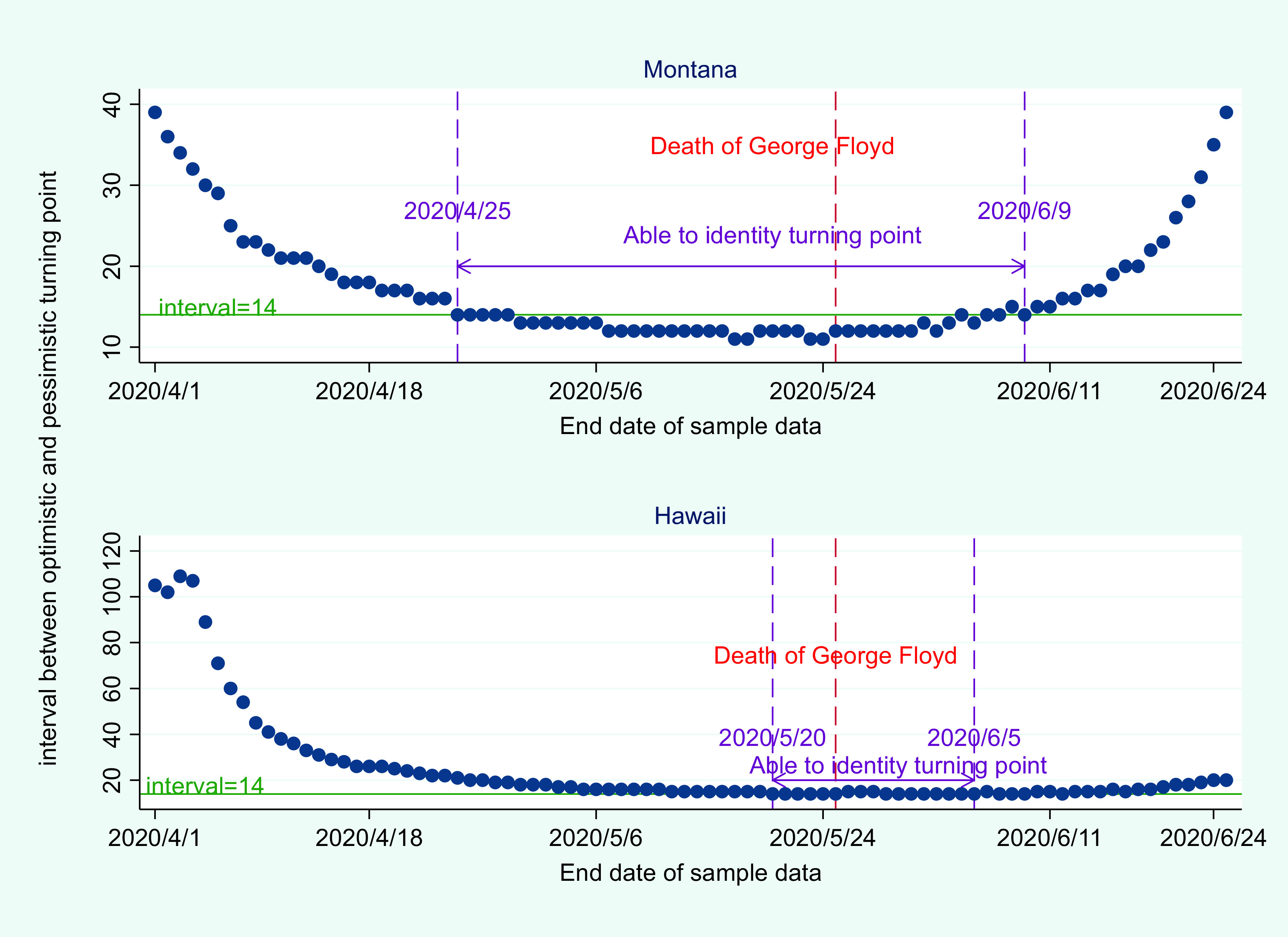
Selection of sample size and its impact on interval prediction.

To test the robustness of the methodology in this study, we change the sample size and evaluate its impact on the prediction of the turning point. To be more specific, the start date of sample data for forecasting is fixed in this study, which is January 21, 2020. And the end date of sample data ranges between April 1, 2020, and June 25, 2020. As is shown in Figure 5, the interval between optimistic and pessimistic turning points is relatively high when the end date of the sample data is April 1, 2020. As the end date of sample data approaches May 25, 2020, the interval is shrinking steadily.

The implication is that uncertainty of the coronavirus pandemic is fading away in both Montana and Hawaii before May 25, 2020. However, uncertainty decreases at different rates for Montana and Hawaii. For Montana, it is possible to identify the first turning point of coronavirus in Montana ever since April 25, 2020 (see Figure 5). For Hawaii, it is not until May 20, 2020 that we can identify the first turning point.

It should be noted that the Black Lives Matter movement, which began on May 26, 2020, did have an impact on prediction of coronavirus in the United States. As is shown in Figure 5, it is becoming more difficult to identify the turning point of coronavirus in Montana and Hawaii after June 9, 2020, and June 5, 2020, respectively. This shows both the validity and limitations of the method suggested in this study. Because we are able to identify the first turning point for both Montana and Hawaii using different sample sizes, this proves the validity of the method. However, as mentioned before, the models used here depend on a closed nonmobile population. Due to the large-scale protests in the United States, the model does not apply to the period 2 wk after the death of George Floyd. In other words, the model suggested here is able to predict the first turning point of coronavirus, but might fail if there is a second wave of coronavirus caused by large scale protests.

**FIGURE 6:**
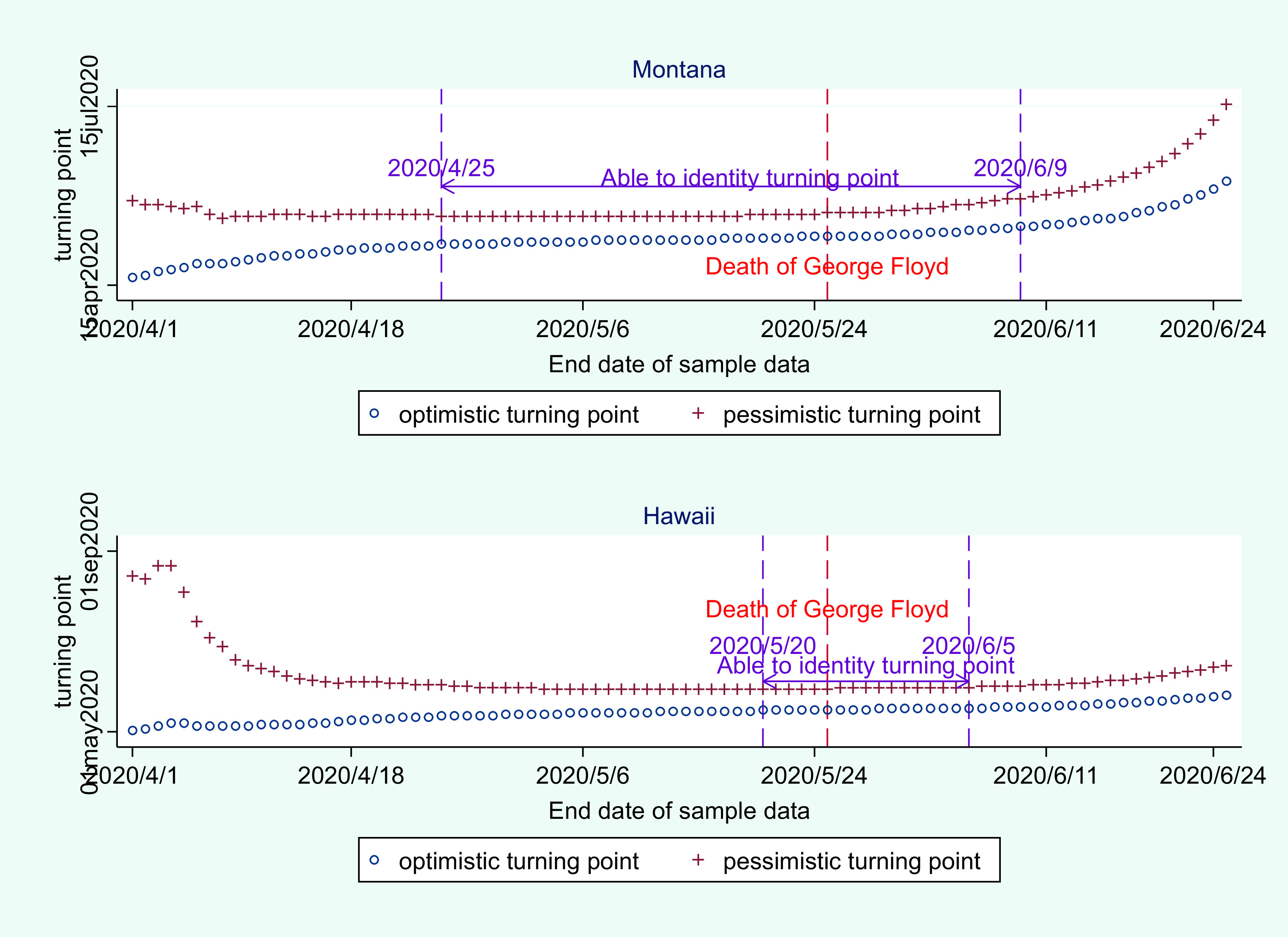
Selection of sample size and its impact on prediction of optimistic and pessimistic turning points.

Further analysis shows that the prediction of the turning point is very stable. As is shown in Figure 6, using sample data between January 21, 2020, and April 25, 2020, the predicted turning point for Montana lies between May 6, 2020, and May 20, 2020. Using sample data between January 21, 2020, and May 25, 2020, the predicted turning point for Montana lies between May 10, 2020, and May 22, 2020. When the end date of sample data ranges between May 20, 2020, and May 25, 2020, the predicted turning point for Hawaii remains the same, which lies between May 16, 2020, and May 30, 2020.

Therefore, it can be concluded that the prediction of the turning point for Montana and Hawaii is robust. The intuition behind the analysis is that 4 predicted trajectories of COVID-19 should converge when the pandemic reaches its first peak in a specific state. As is shown in Figure 6, the optimistic and pessimistic turning point converges to a stable interval, which provides a basis for judging the actual turning point of the epidemic in the United States.

However, it should be noted that when the end date of sample data is chosen 2 wk after George Floyd’s death, the estimated optimistic and pessimistic turning point does not converge to a stable interval. In other words, the model suggested here is valid in determining the first turning point when lockdown countermeasures did not change much. Once lockdown measures become ineffective due to nationwide protests in the United States, the model is invalid in predicting the second turning point of coronavirus.

## DISCUSSION

Under the stress of economic stagnation, many states in the United States have reopened their economies. However, from the analysis of this study, the outbreak has not been sufficiently controlled. Based on the real pandemic data, it is estimated that only Montana and Hawaii have reached their first peaks. It is still too early to forecast the first turning points for the majority of states.

In addition, using data of the number of confirmed cases from January 21, 2020, to May 25, 2020, in the United States, it is predicted that 75% of regions will not reach the first peak of coronavirus before August 2, 2020. Furthermore, mass protests have caused the pandemic to rebound and made it more difficult to forecast the second turning point of coronavirus. Therefore, COVID-19 will continue to last for a while in the United States. Local governments need to be cautious if there is a timeline for further work resumption.

## CONCLUSIONS

Using the logistic and Gompertz models, we put forward a methodology to detect the possible turning point of the coronavirus pandemic in the United States. The method takes into account the real-time information of the confirmed cases, which can provide a plausible prediction of the first turning point. By changing the sample size of projection, we also proved the robustness of the methodology mentioned above. This methodology can provide a credible prediction of the actual turning point for different regions in the United States. This study may be further extended to forecast the outbreak in other countries. However, it should also be noted that the model proposed here is valid for a closed nonmobile population. Therefore, the model is valid in predicting the first turning point of coronavirus using sample data before May 26, which is the starting date for Black Lives Matter movement. But it might fail in predicting the second turning point of coronavirus caused by the nationwide protests in the United States.

The implication is that the coronavirus pandemic in the United States is out of control in most regions. The reason is that the pandemic has not peaked in most regions. Although Montana and Hawaii used to reach their first peaks, there is a rebound of the pandemic in the 2 states due to massive protests. For the majority of states, people need to keep countermeasures in place, at least through August 2, 2020. Because the pandemic has not peaked in most regions of the United States, it is still crucial to take essential social distancing rules while reviving the economies. The model in this study is valid for a closed nonmobile population. Thus, it can be used to forecast the second turning point of coronavirus for states that are closing again.
